# Supplementary material for: Spatial single-cell mass spectrometry defines zonation of the hepatocyte proteome
Source: Nat Methods. 2023 Oct 2;20(10):1530–6. doi: 10.1038/s41592-023-02007-6 (PMC10555842; doi:10.1038/s41592-023-02007-6)
Supplement: Supplementary file 1 — Reporting Summary [file 41592_2023_2007_MOESM1_ESM.pdf]

nature portfolio

Corresponding author(s): Matthias Mann

Last updated by author(s): Jun 16, 2023

Reporting Summary

Nature Portfolio wishes to improve the reproducibility of the work that we publish. This form provides structure for consistency and transparency in reporting. For further information on Nature Portfolio policies, see our Editorial Policies and the Editorial Policy Checklist.

Statistics

For all statistical analyses, confirm that the following items are present in the figure legend, table legend, main text, or Methods section.

n/a	Confirmed	
☐	☒	The exact sample size (*n*) for each experimental group/condition, given as a discrete number and unit of measurement
☐	☒	A statement on whether measurements were taken from distinct samples or whether the same sample was measured repeatedly
☐	☒	The statistical test(s) used AND whether they are one- or two-sided<br>*Only common tests should be described solely by name; describe more complex techniques in the Methods section.*
☐	☒	A description of all covariates tested
☐	☒	A description of any assumptions or corrections, such as tests of normality and adjustment for multiple comparisons
☐	☒	A full description of the statistical parameters including central tendency (e.g. means) or other basic estimates (e.g. regression coefficient) AND variation (e.g. standard deviation) or associated estimates of uncertainty (e.g. confidence intervals)
☐	☒	For null hypothesis testing, the test statistic (e.g. *F*, *t*, *r*) with confidence intervals, effect sizes, degrees of freedom and *P* value noted<br>*Give P values as exact values whenever suitable.*
☒	☐	For Bayesian analysis, information on the choice of priors and Markov chain Monte Carlo settings
☒	☐	For hierarchical and complex designs, identification of the appropriate level for tests and full reporting of outcomes
☐	☒	Estimates of effect sizes (e.g. Cohen's *d*, Pearson's *r*), indicating how they were calculated

Our web collection on statistics for biologists contains articles on many of the points above.

Software and code

Policy information about availability of computer code

Data collection	Commercially available: Perkin Elmer Harmony 4.9, Biological Image Analysis Software BIAS v2022-02-02 (Single-Cell Technologies Ltd., ref 4 and acknowledgements), Leica LMD 8.2, Leica LMD beta 10, Bruker HyStar 6.0, timsControl 3.0.20, Evosep One RCNet Driver 2.2.74.0. Open source: ashlar python API (ref 26)
Data analysis	FragPipe 18.0 (ref 29), MSFragger 3.5 Philosopher 4.4.0, EasyPQP 0.1.32, DIA-NN 1.8.1 (ref 30), MaxQuant 2.1.3.0, R 4.2.1 [packages: WebGestaltR 0.4.4, ggridges 0.5.4, RColorBrewer 1.1-3, sf 1.0-9, XML 3.99-0.12, rstatix 0.7.0, MASS 7.3-57, devtools 2.4.5, org.Mm.eg.db 3.15.0, AnnotationDbi 1.58.0, IRanges 2.30.1, pheatmap 1.0.12, limma 3.52.4, viridis 0.6.2, viridisLite 0.4.1, ggrepel 0.9.1, forcats 0.5.2, stringr 1.4.1, dplyr 1.0.10, purrr 0.3.5, readr 2.1.3, tidyr 1.2.1, tibble 3.1.8, ggplot2 3.3.6, tidyverse_1.3.2], RStudio 2022.07.2, Python 3.8.11 [packages: Pillow 9.0.0, Numpy 1.4.2, pandas 1.22.3, scikit-learn 1.0.2, Yellowbrick 1.5]. Custom open source: py_diAID (ref 28), RefQuant (ref 11).

For manuscripts utilizing custom algorithms or software that are central to the research but not yet described in published literature, software must be made available to editors and reviewers. We strongly encourage code deposition in a community repository (e.g. GitHub). See the Nature Portfolio guidelines for submitting code & software for further information.

Data

Policy information about availability of data

All manuscripts must include a data availability statement. This statement should provide the following information, where applicable:
- Accession codes, unique identifiers, or web links for publicly available datasets
- A description of any restrictions on data availability
- For clinical datasets or third party data, please ensure that the statement adheres to our policy

The mass spectrometry proteomics data have been deposited to the ProteomeXchange Consortium via the PRIDE 34 partner repository with the ID PXD038699. Imaging data has been deposited to BioImages with the accession number S-BIAD596.

Human research participants

Policy information about studies involving human research participants and Sex and Gender in Research.

Reporting on sex and gender	NA
Population characteristics	NA
Recruitment	NA
Ethics oversight	NA

Note that full information on the approval of the study protocol must also be provided in the manuscript.

Field-specific reporting

Please select the one below that is the best fit for your research. If you are not sure, read the appropriate sections before making your selection.

☒ Life sciences ☐ Behavioural & social sciences ☐ Ecological, evolutionary & environmental sciences

For a reference copy of the document with all sections, see nature.com/documents/nr-reporting-summary-flat.pdf

Life sciences study design

All studies must disclose on these points even when the disclosure is negative.

Sample size	Five-shape proteomes: n = 5 mice, 230 samples. Single-shape proteomes: n = 3 mice, 459 samples; one validation mouse with 54 additional samples. No sample size calculations were performed, but estimated to full 10 days of full time MS measurements (> samples).
Data exclusions	Single-shape samples were excluded if the number of proteins was below 806 or above 3362 proteins (median number of proteins - 1.5 SD or + 3 SD; discarded 42, kept 418). Four samples were removed due to their outlier position in the principal component analysis (discarded 4: m3B_14_target8, m3B_40_target4, m4A_58_target8, m4A_67_target4; kept 414). Eight samples were removed due to their cell sizes larger than 1350 µm2 (kept 406).
Replication	Each single-cell slice per mouse was treated as an individual replicate, thus amounting to a total of 400 hepatocytes and 6 arterioles. Batch correction was applied in an unsupervised way via RefQuant in relation to the reference proteome channel. Fifty-four additional samples from a separate mouse were used as a validation of the data.
Randomization	Allocation of samples was performed randomly and in an unsupervised way. Of all segmented hepatocytes, every 15th, 20th or 25th (depending on the total number of hepatocytes per section to reach wide coverage across one section) was cut and measured. The samples of one mouse were prepared and measured in one batch (in the order m5C, m4A, m3B, m1A) as samples should be as fresh as possible when analyzed by LC-MS/MS.
Blinding	Investigators were not blinded due to the study design, that is experimentally verifying ground truth to confirm robustness of the method. Several steps were unsupervised to eliminate observer bias: mice were sacrificed in a random order, selection of hepatocytes was random (every 15th, 20th or 25th of all segmented hepatocytes depending on total number of cells per section), samples were excluded according to a calculated cutoff (see data exclusions). -- Exclusion based on PCA position (n = 4 of 414 samples) was not biased.

Reporting for specific materials, systems and methods

We require information from authors about some types of materials, experimental systems and methods used in many studies. Here, indicate whether each material, system or method listed is relevant to your study. If you are not sure if a list item applies to your research, read the appropriate section before selecting a response.

Materials & experimental systems

n/a	Involved in the study	
☐	☒	Antibodies
☒	☐	Eukaryotic cell lines
☒	☐	Palaeontology and archaeology
☐	☒	Animals and other organisms
☒	☐	Clinical data
☒	☐	Dual use research of concern

Methods

n/a	Involved in the study	
☒	☐	ChIP-seq
☒	☐	Flow cytometry
☒	☐	MRI-based neuroimaging

Antibodies

Antibodies used	Anti-e cadherin coupled to Alexa Fluor 555 (BD 560064) used at 1:100<br>Anti-glutamine synthase (rabbit, Abcam ab176562) used at 1:200<br>Anti-rabbit nanobody coupled to Alexa Fluor 647 (Chromotek srbAF647-1-100) used at 1:500
Validation	BD 560064: verified by manufacturer with Western blotting using purified mouse E-Cadherin and overpression of human E-Cadherin in 293F cells<br>ab176562: validated by manufacturer with Western blotting, and a HAP1 KO cell line

Animals and other research organisms

Policy information about studies involving animals; ARRIVE guidelines recommended for reporting animal research, and Sex and Gender in Research

Laboratory animals	Pathogen-free male and female 10-week-old C57BL/6J-rj mice were purchased from Janvier (France) and maintained at the appropriate biosafety level under constant temperature and humidity conditions with a 12h light cycle. Animals were allowed food and water ad libitum. All experiments were performed on 12- or 13-week-old wild-type mice.
Wild animals	No wild animals were used in this study.
Reporting on sex	Only male mice were used due to the study design, i.e. validation of a method.
Field-collected samples	No field-collected samples were used in this study.
Ethics oversight	Animal handling and organ withdrawal were performed in accordance with the governmental and international animal welfare guidelines and ethical oversight by the local government for the administrative region of Upper Bavaria (Germany), registered under ROB-55.2-2532.Vet_02-16-208.

Note that full information on the approval of the study protocol must also be provided in the manuscript.
